# The complete chloroplast genome of *Ficus pumila*, a functional plant in East Asia

**DOI:** 10.1080/23802359.2022.2031328

**Published:** 2022-02-03

**Authors:** DanYang Liang, Huqiang Lv, Jianxiong Liao

**Affiliations:** a Colle ge of Tourism & Landscape Architecture (College of Plant and Ecological Engineering), Guilin University of Technology, Guilin, P. R. China; b Guangxi Key Laboratory of Plant Conservation and Restoration Ecology in Karst Terrain, Guangxi Institute of Botany, Guangxi Zhuang Autonomous Region and Chinese Academy of Sciences, Guilin, P. R. China; c Federation of Supply and Marketing Cooperatives, Xi'an Research Institute of Chinese Lacquer Under All China, Xi'an, P. R. China

**Keywords:** *Ficus pumila*, chloroplast genome, Illumina sequencing, functional plant

## Abstract

*Ficus pumila* L. is a climbing fig commonly used as an ornamental plant. In this study, we sequenced and assembled the complete chloroplast genome of *F. pumila*. The complete chloroplast genome of *F. pumila* is 160,248 bp in length which includes a pair of inverted repeats (IRs) of 25,871 bp separated by a large single-copy (LSC) region of 88,405 bp and a small single-copy (SSC) region of 20,101 bp. The overall guanine–cytosine (GC) content of *F. pumila* cp genome is 35.98%, while the corresponding values of LSC, SSC, and IR sequences are 33.65, 29.05, and 42.65%, respectively. The phylogenetic tree was shown to be consistent with the traditional morphology-based taxonomy of *Moraceae*. Five plants from the genus *Ficus* formed a well-supported monophyletic clade with 100% bootstrap value, and *F. pumila* is closely related to *F. hirta*, *F. carica*, and *F. racemosa*, with a support value of 97%. The complete chloroplast of *F. pumila* contributes to the growing number of chloroplast genomes for phylogenetic and evolutionary studies in Moraceae.

*Ficus pumila* Linnaeus (1753) (Linne 1753) is a climbing fig commonly used as an ornamental plant. It is a root climbing evergreen vine attaching to rocks, walls, tree trunks by means of exudations from the aerial roots (Kaur 2012). It is reported to be native to East Asia, specifically South China through to Malaysia, but now it is cultivated in numerous countries around the world. The plant is resistant to drought and can also grow under extreme shadow. Therefore, *F. pumila* has great potential in ecological restoration and vertical greening, and the values of *F. pumila* in ecological restoration and greening should also attract special attention (Qi et al. 2021). Moreover, the plant is believed to have medicinal value. For example, the fruit containing triterpenoids and sterols are used as tonic medicament, antitumor and anti-inflammatory in Chinese folk (Kitajima et al. 2000). The leaves containing flavonoid glycosides are traditionally used by some Okinawan elders as beverage or herbal medicines to treat dizziness, diabetes, neuralgia and hypertension. (Liang et al. 2012). Therefore, there is an urgent need to obtain genetic and genomic information to advance its systematic research and develop its conservation value of *F. pumila*.

In this study, the chloroplast genome of *F. pumila* was sequenced, and its molecular phylogeny and genetic information were determined. The sample of *F. pumila* was collected from the Guangxi Institute of Botany (GPS: 25°4'46" N, 110°17'52" E) in Guilin, China. The collection of plant materials are in accordance with local regulations and obtain the permission of local authorities. A specimen was deposited at the herbarium of Guangxi Institute of Botany (http://www.gxib.cn/spIBK/, Z. C. Lu, email: zhaocenlu@163.com) under the voucher number IBK00435049. Total genomic DNA was extracted using a modified CTAB (Cetyltrimethyl Ammonium Bromide) method (Doyle and Doyle 1987). A total of 6 G raw data from Illumina Hiseq Platform were screened. De novo genome assembly and annotation were conducted by GetOrganelle (Jin et al. 2020) and CPGAVAS2 (Shi et al. 2019), respectively. The complete cp genome was deposited in GenBank (accession number: MZ351203).

The complete chloroplast genome of *F. pumila* is 160,248 bp in length which includes a pair of inverted repeats (IRs) of 25,871 bp separated by a large single-copy (LSC) region of 88,405 bp and a small single-copy (SSC) region of 20,101 bp. The overall guanine–cytosine (GC) content of *F. pumila* cp genome is 35.98%, while the corresponding values of LSC, SSC, and IR sequences are 33.65, 29.05, and 42.65%, respectively.

In order to explore the phylogenetic position of *F. pumila* with other *Moraceae*, total 14 complete chloroplast genomes published species and one outgroup *Antiaris toxicaria* (NC042884) were obtained from Genbank. A total of 87 coding sequences were aligned using MUSCLE (Edgar 2004). Phylogenetic analysis was conducted using maximum likelihood (ML) method by RAxML version 8.2.12 (Stamatakis 2014) with 100 bootstrap replicates. The phylogenetic analysis (Figure 1) showed that five plants from the genus *Ficus* formed a well-supported monophyletic clade with 100% bootstrap value, and *F. pumila* is closely related to *Ficus hirta*, *Ficus carica*, and *Ficus racemosa*, with a support value of 97%. The complete chloroplast of *F. pumila* contributes to the growing number of chloroplast genomes for phylogenetic and evolutionary studies in *Moraceae*.

**Figure 1.**
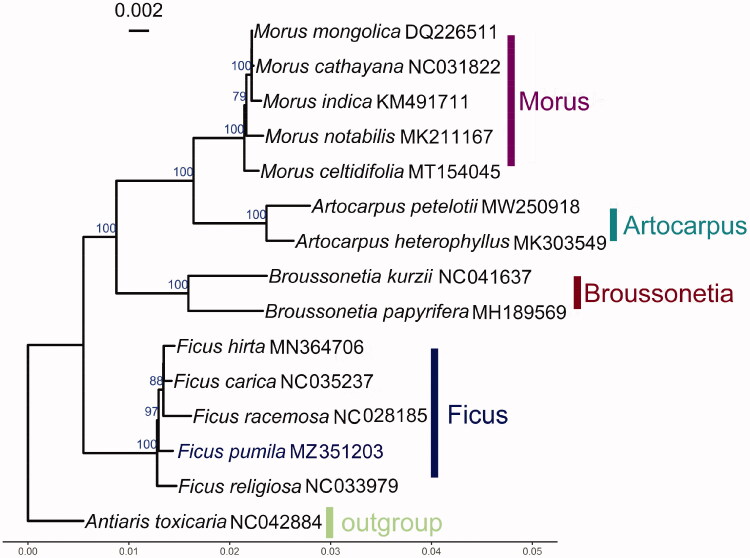
ML phylogenetic tree based on chloroplast gene sequences of *Ficus pumila* and other 14 species. Numbers in the nodes are the bootstrap values from 100 replicates. Bootstrap support values >75% are indicated next to the branches.

## Data Availability

The genome sequence data that support the findings of this study are openly available in GenBank of NCBI at (https://www.ncbi.nlm.nih.gov/) under the accession no. MZ351203. The associated BioProject, SRA, and BioSample numbers are PRJNA763858, SRX12204792, and SAMN21454087 respectively. Treefile of 15 species and genes for phylogenetic analysis were deposited at Figshare: https://doi.org/10.6084/m9.figshare.16628617.v1.
